# Dehydrocostus Lactone Suppresses Dextran Sulfate Sodium-Induced Colitis by Targeting the IKKα/β-NF-κB and Keap1-Nrf2 Signalling Pathways

**DOI:** 10.3389/fphar.2022.817596

**Published:** 2022-03-07

**Authors:** Yun Yuan, Qiongying Hu, Lu Liu, Fan Xie, Luyao Yang, Yuchen Li, Chuantao Zhang, Hongqing Chen, Jianyuan Tang, Xiaofei Shen

**Affiliations:** ^1^ Hospital of Chengdu University of Traditional Chinese Medicine, Chengdu, China; ^2^ College of Medical Technology, Chengdu University of Traditional Chinese Medicine, Chengdu, China; ^3^ Department of Laboratory Medicine, Hospital of Chengdu University of Traditional Chinese Medicine, Chengdu, China; ^4^ College of Pharmacy, Chengdu University of Traditional Chinese Medicine, Chengdu, China; ^5^ Department of Respiratory Medicine, Hospital of Chengdu University of Traditional Chinese Medicine, Chengdu, China

**Keywords:** colitis, dehydrocostus lactone, inflammation, macrophages, IKKα/β-NF-κB pathway, Keap1-Nrf2 pathway

## Abstract

Dehydrocostus lactone (DCL) is a major sesquiterpene lactone isolated from *Aucklandia lappa Decne*, a traditional Chinese herbal medicine that used to treat gastrointestinal diseases. This study aimed to examine the therapeutic effects of DCL on dextran sulfate sodium (DSS)-induced colitis with a focus on identifying the molecular mechanisms involved in DCL-mediated anti-inflammatory activity in macrophages. First, oral administration of DCL (5–15 mg/kg) not only ameliorated symptoms of colitis and colonic barrier injury, but also inhibited the expression of proinflammatory cytokines and myeloperoxidase in colon tissues in DSS-challenged mice. Furthermore, DCL also exhibited significant anti-inflammatory activity in LPS/IFNγ-stimulated RAW264.7 macrophages. Importantly, DCL significantly suppressed the phosphorylation and degradation of IκBα and subsequent NF-κB nuclear translocation, and enhanced the nuclear accumulation of Nrf2 in LPS/IFNγ-treated RAW264.7 cells. Mechanistically, DCL could directly interact with IKKα/β and Keap1, thereby leading to the inhibition of NF-κB signalling and the activation of Nrf2 pathway. Furthermore, DCL-mediated actions were abolished by dithiothreitol, suggesting a thiol-mediated covalent linkage between DCL and IKKα/β or Keap1. These findings demonstrated that DCL ameliorates colitis by targeting NF-κB and Nrf2 signalling, suggesting that DCL may be a promising candidate in the clinical treatment of colitis.

## Highlights

- DCL inhibits DSS-induced colitis in mice
- DCL shows anti-inflammatory activity in LPS/IFNγ-stimulated macrophages
- DCL treatment results in inhibition of NF-κB pathway and activation of Nrf2 signalling
- DCL interacts with IKKα/β and Keap1 to inhibit NF-κB pathway and activate Nrf2 signalling

## Introduction

Ulcerative colitis (UC) is a major type of inflammatory bowel disease (IBD) with unspecific colorectal inflammation. UC is characterized by recurrent mucositis starting in the rectum and extending to the proximal colon (Feuerstein et al., 2019). The pathogenesis of UC is multifactorial and may be due to genetic predisposition, epithelial barrier defects, imbalanced immune responses, and environmental factors (Ordás et al., 2012). Currently, the clinical therapy for patients with UC involves 5-aminosalicylic acid (5-ASA), sulfasalazine (SASP), corticosteroids, thiopurines, and calcineurin inhibitors (Harbord et al., 2017). Although these commercial drugs have been used universally, they have possibilities of causing severe side effects. For example, it has been reported that SASP treatment resulted in male infertility, nephrotoxicity, and hepatotoxicity (Linares et al., 2011). Therefore, discovering potential and highly efficient anti-UC drugs with few adverse body reactions is extremely important for UC clinical therapy.

Currently, UC is thought to be directly actuated by immune dysfunction of the intestinal mucosa (Pithadia and Jain, 2011). Macrophages, the major immune cells, play pivotal roles in intestinal homeostasis by regulating the initiation, maintenance, and resolution of inflammation. During UC, macrophages can be activated by a series of proinflammatory stimuli, including bacterial lipopolysaccharide (LPS) and interferon-γ (IFNγ) (Na et al., 2019). Activated macrophages then synthetize excessive reactive oxygen species (ROS) and proinflammatory cytokines (such as interleukin [IL-6] and tumour necrosis factor-α [TNF-α]) *via* the activation of nuclear factor-κB (NF-κB) and mitogen-activated protein kinase (MAPK) signalling pathways (Wu et al., 2020). These excessive proinflammatory factors not only trigger inflammatory responses by recruiting and activating neutrophils and lymphocytes but also result in injury to the colonic mucosa (Yao et al., 2019). Therefore, suppressing the population of macrophages or their biological functions may act as a novel therapeutic strategy for UC.

*Aucklandia lappa* Decne, a plant belonging to the Asteraceae family, is a multipurpose species that is also used in medicine, food, fodder, and perfume. In China, the National Health Commission has approved *Aucklandia lappa* Decne as a functional food (Zhuang et al., 2021). Furthermore, *Aucklandia lappa* Decne is widely used in traditional Chinese medicine or folk medicine for the treatment of abdominal distention, vomiting, diarrhoea, and dysentery tenesmus (Guo et al., 2014; Zhuang et al., 2021). Therefore, *Aucklandia lappa* Decne is considered a medicinal and edible homologous plant (Nadda et al., 2020; Zhuang et al., 2021). Dehydrocostus lactone (DCL) is a natural sesquiterpene lactone that is considered as the main bioactive element in *Aucklandia lappa* Decne (Li et al., 2020). Previous studies have demonstrated that DCL has potent effects in various pathologies, such as anticancer, anti-inflammatory, antioxidative stress, and antimycobacterial activities (Cantrell et al., 1998; Choi et al., 2009; Wang J et al., 2017; Xiong et al., 2021). A recent study has demonstrated that oral administration of DCL ameliorates DSS-induced colitis *via* the reduction of proinflammatory cytokines and the suppression of the IL-6/STAT3 signalling pathway (Zhou et al., 2020). However, the precise molecular mechanism of DCL-mediated anti-colitis and anti-inflammatory activity is still unknown. In the present study, we investigated the anti-inflammatory and therapeutic effects of DCL in DSS-induced colitis in mice and LPS/IFNγ-stimulated murine RAW264.7 macrophages, respectively. Furthermore, we also investigated the underlying mechanism of DCL-mediated anti-inflammatory activity, which may provide a scientific basis for the development of anti-inflammatory agents for UC therapy.

## Materials and Methods

### Reagents

DCL (Cat# PU0624-0025) was purchased from Push Bio-Technology Co., Ltd. (Chengdu, China). The purity of DCL is greater than 98%. DSS (M.W: 36,000–50,000; Cat# MB5535) and salicylazosulfapyridine (SASP, Cat# MB5634) were obtained from Meilun Biotechnology (Dalian, China). LPS extracted from *E. coli* 055:B5 (Cat# L6529) was obtained from Sigma-Aldrich (Shanghai, China). Recombinant murine IFNγ (Cat# 50709-MNAH) was provided by SinoBiological (Beijing, China). BAY 11-7082 (Cat# S1523) and curcumin (Cat# SC0299) were purchased from Beyotime (Shanghai, China). PD98059 (Cat# S1177), SB203580 (Cat# S1076), and SP600125 (Cat# S1460) were provided by Selleck (Shanghai, China). Primary antibodies against COX-2 (Cat# 12375-1-AP), iNOS (Cat# 18985-1-AP), β-Actin (Cat# 20536-1-AP), α-Tubulin (Cat# 11224-1-AP), GAPDH (Cat# 10494-1-AP) and HRP-conjugated affinipure goat antibody (Cat# SA00001-2) were purchased from ProteinTech (Wuhan, China). Primary antibodies against IKKα/β (Cat# AF2221), phospho-IKKα/β (p-IKKα/β) (Cat# AF5839), IκBα (Cat# AF1282), phospho-IκBα (p-IκBα) (Cat# AF 1870), ERK1/2 (Cat# AF1051), phospho-ERK1/2 (Cat# AF5818), JNK1/2/3 (Cat# AF1048), phospho-JNK1/2/3 (Cat# AF1762), P38 (Cat# AF7668), phospho-P38 (Cat# AF5887) were purchased from Beyotime (Shanghai, China). Primary antibodies against Keap1 (Cat# AF5266), Nrf2 (Cat# AF7006) and HO-1 (Cat# AF5393) were purchased from Affinity Biosciences (Cincinnati, OH, United States). Primary antibodies against CD68 (Cat# GB113109), myeloperoxidase (MPO, Cat# GB11224), TNF-α (Cat# GB11188), zonula occluden-1 (ZO-1, Cat# GB111402), Occludin (Cat# GB111401) were purchased from Servicebio (Wuhan, China).

### Mice

ICR mice (SPF grade, 6–8 weeks, 20–22 g, male) were purchased from Chengdu Dossy Experimental Animals Co., Ltd. (Chengdu, China). Mice were kept in the plastic cages filled with poplar wood shavings on a 12-h light/dark cycle at 25 ± 1°C, and fed with standard food and water. Mice were adapted at least 7 days before the experiment. All mice were divided into six groups, containing normal group, DSS group, SASP group, DCL-low-dose (DCL-L) group, DCL-middle-dose (DCL-M) group, DCL-high-dose (DCL-H) group (7 mice/group). All experiments were approved by related ethical regulations of Chengdu University of Traditional Chinese Medicine and are in agreement with the guidelines for the proper use of animals in biomedical research (2021-09).

### Induction of Colitis and Treatments

Mice from DSS group, SASP group, DCL-L group, DCL-M group, and DCL-H group were orally administrated with 4% of DSS dissolved in double distilled water *ad libitum* from day 2 to day 8. Mice from SASP (50 mg/kg/d), DCL-L (5 mg/kg/d), DL-M (10 mg/kg/d), and DL-H (15 mg/kg/d) group were intragastrically administrated with the indicated drugs once a day from day 1 to day 8. Mice from normal group and DSS group were administrated with the vehicle (0.5% sodium carboxymethyl cellulose). The body weights were recorded during day 2 to day 8.

### Histological Assessments of Colon Tissues

Two hours after the last administration, mice were sacrificed by cervical dislocation, and the sleep was removed and weighed. Next, the colon tissues were isolated and the length of colon was measured. Subsequently, colons were fixed with 4% paraformaldehyde overnight, embedded in paraffin. After hematoxylin and eosin (HE) staining, the specimens were observed under the microscope and assessed histopathological score, as described (Yang et al., 2019). Seven independent parameters were measured: the extent of inflammation (0, none; 1, mucosa; 2, mucosa and submucosa; 3, mucosa, submucosa and muscle layer; 4, transmural), infiltration neutrophils and lympho-histiocytes (0, none; 1, focal; 2, multifocal; 3, diffuse), crypt damage (0, none; 1, basal 1/3; 2, basal 2/3; 3, entire crypt damage; 4, crypt damage and ulceration), crypt abscess (0, none; 1, focal; 2, multifocal), sub-mucosal edema (0, none; 1, focal; 2, multifocal; 3, diffuse), loss of goblet cells (0, none; 1, focal; 2, multifocal; 3, diffuse), reactive epithelial hyperplasia (0, none; 1, focal; 2, multifocal; 3, diffuse). The histopathological scores were determined by adding all scores of the aforementioned parameters.

### Periodic Acid-Schiff (PAS) Staining

The PAS staining of the colon sections was performed using Periodic Acid-Schiff Staining Kit according to manufacturer’s instruction. Briefly, after being deparaffinized, the sections were incubated with periodic acid for 10 min at room temperature (RT), and rinsed with distilled water. Next, the sections were stained with Schiff regent for 30 min at 37°C, rinsed with distilled water. Subsequently, the sections were stained with hematoxylin stain regent for 30 s at 37°C, rinsed with distilled water twice. After dehydration, sections were sealed with neutral balsam on slides. All pictures were acquired from X71 (U-RFL-T) microscope at ×400 magnification. The mean of the integrated optical density (IOD) was analyzed using the ImageJ software (NIH, Bethesda, United States).

### Immunohistochemistry

After being deparaffinized and rehydrated, sections were incubated with sodium citrate buffer (0.01 M, pH = 6.0) for 15 min at 100°C. Then, sections were incubated with 3% H_2_O_2_ to neutralize the endogenous peroxidase. Next, sections were blocked with 10% goat serum for 1 h at RT, and incubated with the specific primary antibodies (dilution of 1:200) overnight at 4°C. The specimens were then incubated with horseradish peroxidase-labelled secondary antibody. The signals were visualized with 3,3′-diaminobenzidine (DAB) reagent. The blue color representing hematoxylin and the brown color representing DAB (positive signal). All pictures were acquired from X71 (U-RFL-T) microscope at ×100, ×400 magnification. The mean of the integrated OD was analyzed using the ImageJ software.

### Cell Culture

Mouse monocyte-macrophage cells RAW264.7 (Cat# C7505) were acquired from Beyotime (Shanghai, China) and maintained in high-glucose Dulbecco’s Modified Eagle Medium (DMEM, Gibco, NY, United States) containing 10% inactivated fetal bovine serum (FBS, PAN biotech, Germany) under standard conditions (37°C with 95% humidity and 5% CO_2_).

ICR Mice were stimulated by intraperitoneal injection of 5% sterile thioglycolate solution (2 ml/mouse) for 3 days before cell isolation. Total primary peritoneal macrophages (PMs) were harvested by washing the peritoneal cavity with DMEM (10 ml/mouse). Cell suspension was centrifuged and suspended in DMEM supplemented with 10% FBS, cells were then seeded into the 96-well plate.

### Cell Viability Assay

RAW264.7 cells (1.5 × 10^4^ cells/100 μl) seeded in 96-well plates were treated with various concentrations of DCL for 2 h, and then stimulated with LPS (0.5 μg/ml) and IFNγ (10 ng/ml) for 24 h. Next, 10 μl of Cell Counting Kit-8 (CCK-8) (AbMole, Shanghai, China) solution was added to each well for 1 h at 37°C. The optical density (OD) at 450 nm was analyzed using a multifunctional microplate reader (FlexStation 3; Molecular Devices).

### Measurement of Nitric Oxide (NO) Production

Briefly, RAW264.7 cells or primary PMs (1.5 × 10^4^ cells/100 μl) were plated in 96-well plates overnight and treated with various positive control drugs and concentrations of DCL prior to stimulation of LPS (0.5 μg/ml) and IFNγ (10 ng/ml) for 24 h. The NO production in cells were detected immediately by using the Griess assay according to the protocol of the manufacturer (Beyotime, Shanghai, China).

### Measurement of Prostaglandin E2 (PGE_2_)

RAW264.7 cells (2×10^5^ cells/500 μl) were plated in 24-well plates overnight and treated with various positive control and concentrations of DCL prior to stimulation of LPS (0.5 μg/ml) and IFNγ (10 ng/ml) for 24 h. The concentrations of PGE_2_ in the cell culture supernatant were determined using an enzyme linked immunosorbent assay (ELISA) kit (R&D Systems, United States) according to manufacturer’s instruction.

### Western Blotting Analysis

Total protein was extracted from cells using radio immunoprecipitation assay lysis (RIPA) buffer supplemented with protease inhibitor (1:100, Cat# K1007; ApexBio, Houston, United States) and the protein concentrations were measured using bicinchoninic acid (BCA) Protein Assay Kit (Cat# P0012S; Beyotime, Shanghai, China). The extraction and isolation of nuclear and cytoplasmic protein were performed according to the method adapted from Bayorh et al. (Bayorh et al., 2006) by using Nuclear and Cytoplasmic Protein Extraction Kit (Beyotime, Shanghai, China). An amount of 20 μg of total proteins was separated on 10% sodium dodecyl sulfate-polyacrylamide gel electrophoresis (SDS-PAGE) and transferred onto polyvinylidene fluoride (PVDF, Thermo Fisher Scientific, United States) membranes. After being blocked with 5% non-fat milk in Tris-HCl buffer saline supplemented with 0.1% Tween-20 (TBST) at RT for 30 min, the membranes were incubated with primary antibodies (dilution of 1:1000) at 4°C overnight. Then, the membranes were washed with TBST for three times and incubated with secondary antibodies (dilution of 1:1000) for 1 h at RT. After being washed with TBST for three times, immunoreactive bands were visualized using electrochemiluminescence (ECL) reagent (Cat# 4AW011, 4A Biotech, Beijing, China) and quantified using ImageJ software.

### Detection of Reactive Oxygen Species (ROS)

RAW264.7 cells (2 × 10^5^ cells/1 ml) were plated in 12-well plates overnight and treated with curcumin (5 μM) and concentrations of DCL prior to stimulation of LPS (0.5 μg/ml) and IFNγ (10 ng/ml) for 8 h. Next, cells were collected and incubated with dichloro-dihydro-fluorescein diacetate (DCFH-DA, 1:1000; Beyotime, Shanghai, China) for 20 min at 37°C. The samples were then detected by using Navios flow cytometer (Beckman Coulter, Brea, United States). The data were analysed using FlowJo 10.4 (FlowJo LLC, Ashland, United States).

### Target Validation

For cellular thermal shift assay (CETSA)*,* RAW264.7 cells (∼10^7^) were suspended with 1 ml of pre-cold PBS containing 1% of protease inhibitor and subjected to three freeze-thaw cycles. The samples were then centrifuged at 12,000 rpm for 10 min at 4°C, and the supernatants were divided equally into two samples, and incubated with DCL (50 μM) or DMSO for 30 min at 37°C. Next, each sample was dispensed to 50 μl of aliquots and heated individually for 3 min at 45°C, 48°C, 51°C, 54°C, 57°C, 60 and 63°C followed by cooling for 3 min at RT, respectively. The heated lysates were centrifuged at 12,000 rpm for 10 min at 4°C to separate the soluble fractions from precipitates. The supernatants were boiled with loading buffer, and analyzed by SDS-PAGE followed by Western blot analysis.

For drug affinity responsive target stability (DARTS) assay, RAW264.7 cells (∼10^7^) were lysed with 600 μl M-PER (Cat# 78503, Thermo Fisher Scientific) supplemented with protease and phosphatase inhibitors. After centrifugation at 12,000 g for 5 min, the supernatant was obtained, and protein content was mixed in 10× reaction buffer [50 mM Tris·HCl (pH 8.0), 50 mM NaCl, 10 mM CaCl_2_]. The solutions were divided equally into two samples, and incubated with DCL (50 μM) or DMSO for 1 h at 25°C. Subsequently, samples were then incubated with pronase (Cat# 10165921001; Roche) (diluted with 1× reaction buffer) for 30 min at 25°C. The samples were boiled with loading buffer, and analyzed by SDS-PAGE followed by Western blotting.

### Qualitative Liquid Chromatography-Mass Spectrometry (LC-MS) Analysis

DCL (10 μM) and DTT (50 μM) were co-incubated in PBS (pH 7.4) for 30 min at 37°C. The sample was then injected into a LC-MS system (Waters Vion^®^ IMS QT, Milford, United States). The MS parameters were optimized as follows: ESI + model, capillary voltage was 3.0 kV, source temperature was 120°C, desolvation temperature was 450°C, cone gas flow was 50 L/h, desolvation gas flow was 800 L/h.

### Molecular Docking

The structure of DCL was obtained from ChemDraw. The conformers of DCL were generated from the Autodock4.2 OPLS2005 force field. The crystallographic structures of IKKβ (PDB: 3QA8) and Keap1 (PDB: 4CXI) were downloaded from Protein Data Bank (PDB). The crystal structures of IKKβ and Keap1 were prepared by the Autodock4.2 Protein Preparation Workflow. The best docking models, with the lowest binding energy were selected, and the structures of the complexes were refined by molecular dynamic simulations of 500 ps, with the OPLS2005 force field. A grid box centered at cysteine 46 (Cys46 or C46) or Cys151 (C151) with a range of 20 Å was generated using Glide. Covalent docking was performed using a Michael addition reaction. The top scored pose was visualized using Pymol.

### Statistics

All data are represented as mean ± SEM (standard error of mean). Student’s t-test or one-way ANOVA followed by Bonferroni test was used to compare two independent variables using GraphPad Prism (Version 8.0.1), and statistically significant differences are indicated as follows: ^#^ *p* < 0.05, ^##^ *p* < 0.01, ^###^ *p* < 0.001, ^####^ *p* < 0.0001, ^*^ *p* < 0.05, ^**^ *p* < 0.01, ^***^ *p* < 0.001, ^****^ *p* < 0.0001.

## Results

### Oral Administration of Dehydrocostus Lactone Protects Mice From DSS-Induced Colitis

**FIGURE 1:**
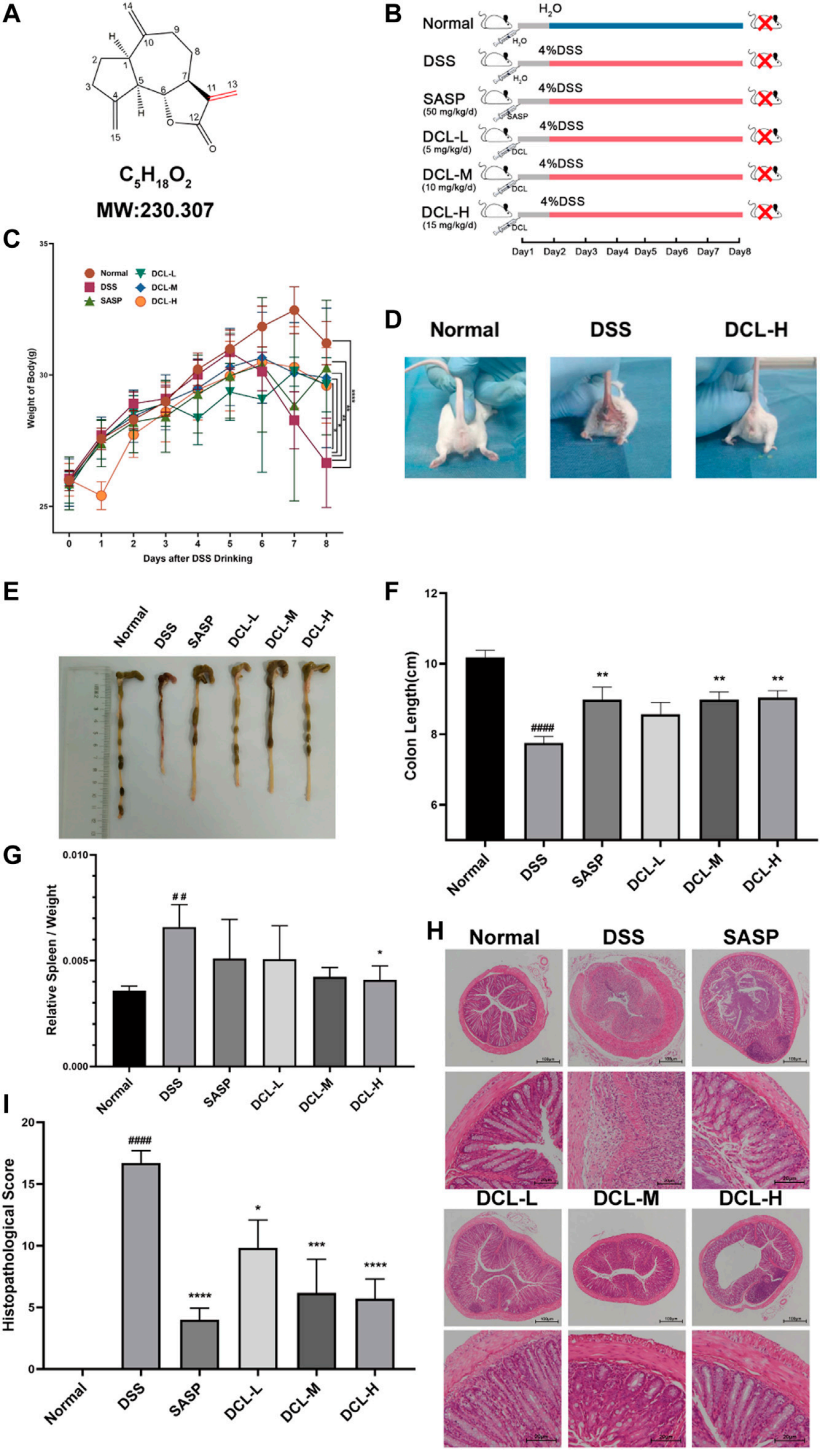
Oral administration of dehydrocostus lactone attenuates symptoms of DSS-induced colitis in mice. **(A)** Structure of DCL. **(B)** Schematic diagram illustrating the experimental procedure. The effects of DCL on body weights **(C)** in DSS-exposed mice. **(D)** Representative photograph of the appearance around crissum of mice from normal, DSS, and DCL-H groups, respectively. Representative photograph of the colon **(E)** and the statistics of colon length **(F)** in each group. The effects of DCL on spleen index **(G)** in DSS-exposed mice. Representative images of HE staining **(H)** and respective histopathological scores **(I)** of colon tissues in each group. Scale bar = 100 or 20 μm. Data are represented as mean ± SEM, *n* = 7. ^#^ *p* < 0.05, ^##^ *p* < 0.01, ^###^ *p* < 0.001, ^####^ *p* < 0.0001 compared to normal group. ^*^ *p* < 0.05, ^**^ *p* < 0.01, ^***^ *p* < 0.001, ^****^ *p* < 0.0001 compared to DSS group.

To examine the therapeutic effect of DCL (Figure 1A) on UC, we generated DSS-induced colitis mice (Figure 1B). As shown in Figure 1C, treatment with DCL (5, 10, and 15 mg/kg) and SASP (50 mg/kg) significantly inhibited weight loss in DSS-challenged mice compared to the vehicle control. Perianal observation showed that DCL or SASP (positive control) treatment remarkedly improved diarrhoea and haematochezia in DSS-treated mice (Figure 1D). Furthermore, DSS administration resulted in colon shortening, which was markedly attenuated by DCL or SASP treatment (Figures 1E,F). Moreover, DCL treatment also reduced the DSS-induced elevation of the spleen index in mice compared to the vehicle control (Figure 1G). In addition, HE staining revealed that treatment with DCL or SASP mitigated the inflammatory injury of colon tissue, including inflammatory cell infiltration, crypt damage, submucosal oedema, goblet cell loss, and reactive epithelial hyperplasia (Figure 1H). Correspondingly, the pathological scores based on a blinded form further supported the observation obtained from HE staining (Figure 1I). Taken together, these findings demonstrated that DCL exhibits a potent therapeutic effect on DSS-induced colitis in mice.

### Dehydrocostus Lactone Improves DSS-Induced Intestinal Epithelial Barrier Injury in Mice

**FIGURE 2:**
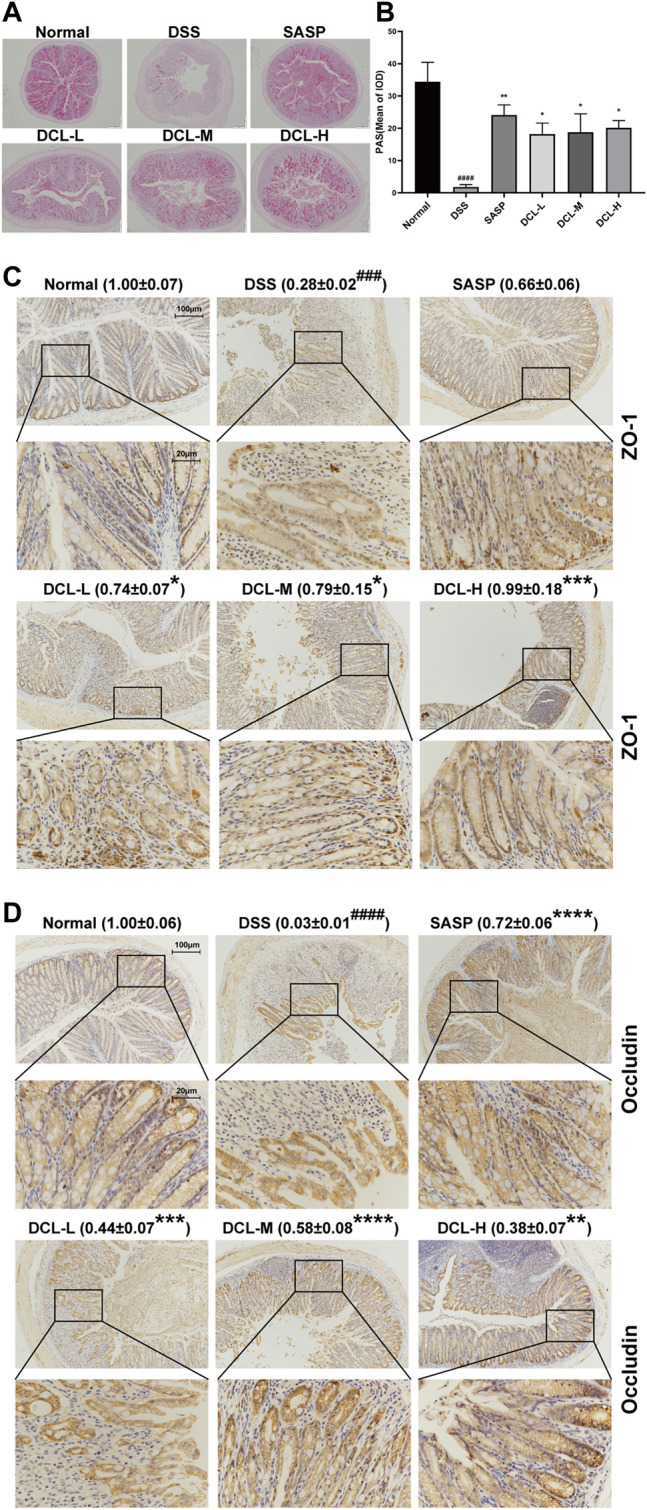
DCL mitigates DSS-induced intestinal barrier injury in mice. Representative PAS images **(A)** and the mean of IOD **(B)** of colon sections in each group. Representative immunohistochemical images and the relative expression of ZO-1 **(C)** and Occludin **(D)** are shown. Scale bar = 200, 100, and 20 μm. The brown areas indicate positive areas. Data are represented as mean ± SEM, *n* = 6. ^#^ *p* < 0.05, ^##^ *p* < 0.01, ^###^ *p* < 0.001, ^####^ *p* < 0.0001 compared to normal group. ^*^ *p* < 0.05, ^**^ *p* < 0.01, ^***^ *p* < 0.001, ^****^ *p* < 0.0001 compared to DSS group.

Impairment of the intestinal mucosal barrier is a hallmark of UC; thus, we assessed the effect of DCL on intestinal barrier functions in DSS-induced colitis. PAS detection showed that DSS administration significantly decreased the numbers of goblet cells in the colon tissues. However, treatment with DCL or SASP rescued the DSS-induced reduction in the number of colonic goblet cells in mice (Figures 2A,B). Next, we investigated whether DCL treatment affects the expression of barrier function-related proteins. As shown in Figures 2C,D, DSS administration resulted in a significant reduction in ZO-1 and occludin, two key proteins involved in intestinal tight junctions. Importantly, DCL or SASP treatment markedly restored the loss of ZO-1 and occludin proteins. Taken together, these results suggested that DCL improves DSS-induced impairment of the intestinal mucosal barrier in mice.

### Dehydrocostus Lactone Alleviates DSS-Induced Intestinal Inflammatory Responses in Mice

**FIGURE 3:**
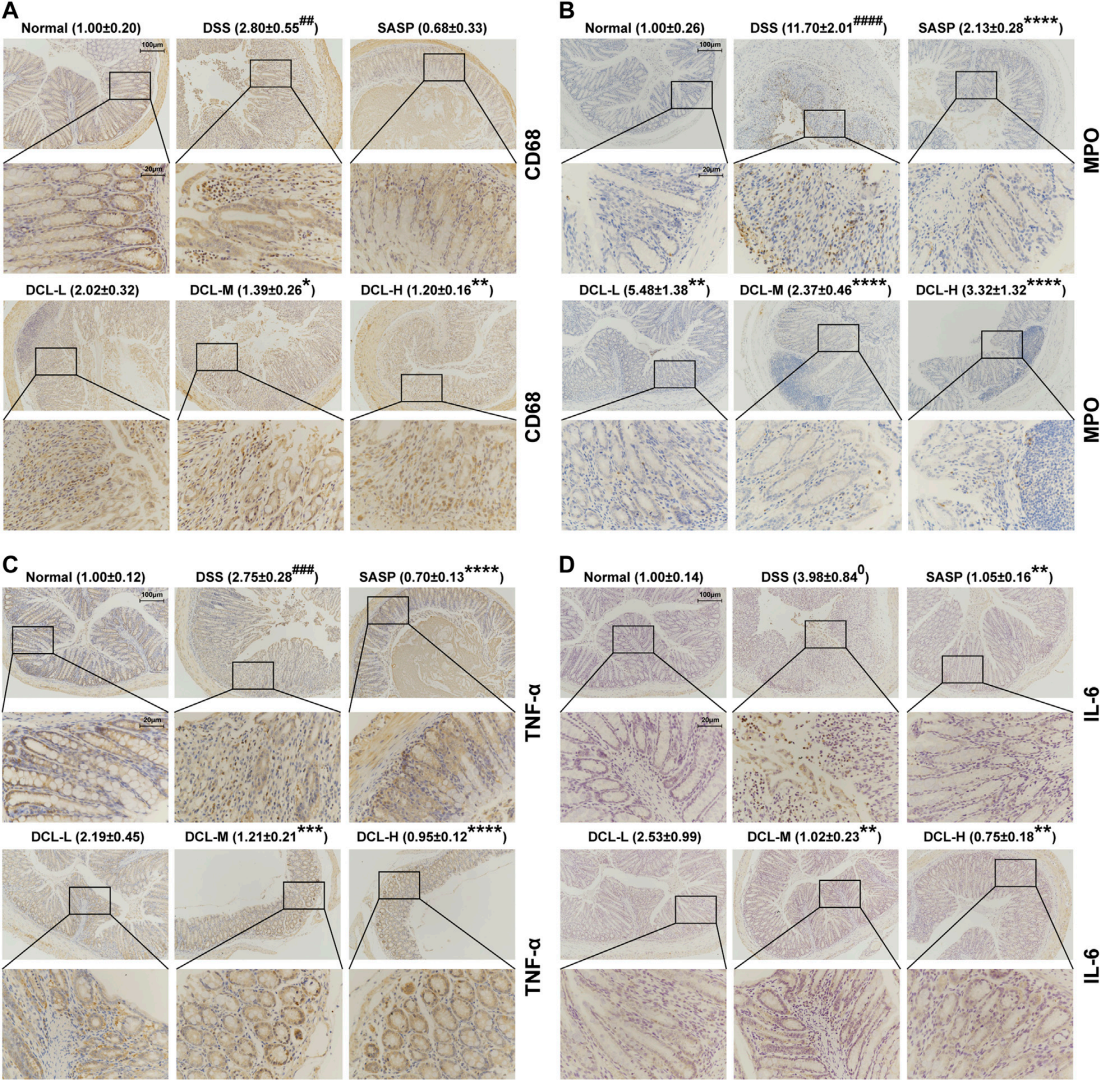
DCL represses intestinal inflammation of DSS-induced colitis in mice. The representative immunohistochemical images and the relative expression of CD68 **(A)** and MPO **(B)** in colon tissues from each group. The effects of DCL on the expression of TNF-α **(C)** and IL-6 **(D)** in DSS- challenged mice. Scale bar = 20 and 100 μm. The brown areas indicate positive areas. Data are represented as mean ± SEM, *n* = 6. ^#^ *p* < 0.05, ^##^ *p* < 0.01, ^###^ *p* < 0.001, ^####^ *p* < 0.0001 compared to normal group. ^*^ *p* < 0.05, ^**^ *p* < 0.01, ^***^ *p* < 0.001, ^****^ *p* < 0.0001 compared to DSS group.

Several immune cells, including macrophages and neutrophils, are recruited into intestinal tissue where these cells are activated in the active phase of UC (Yan et al., 2018). Immunohistochemical analysis showed that DSS induction resulted in high expression of the specific macrophage marker, CD68, while treatment with DCL or SASP notably reduced CD68 expression in colon tissues (Figure 3A). Furthermore, the expression of MPO, a specific marker of neutrophils, was also suppressed by DCL or SASP treatment (Figure 3B) compared to the vehicle control. In addition, these activated immune cells produce excessive proinflammatory cytokines that are involved in the inflammatory response and colon injury (Pervin et al., 2016). As shown in Figures 3C,D, DSS challenge increased proinflammatory cytokines, including TNFα and IL-6, whereas DCL or SASP effectively inhibited the expression of IL-6 and TNFα in colon tissues. These data indicated that DCL attenuates intestinal inflammation in DSS-induced colitis in mice.

### Dehydrocostus Lactone Exhibits Potent Anti-inflammatory Activity in Inhibiting LPS/IFNγ-stimulated Macrophages

**FIGURE 4:**
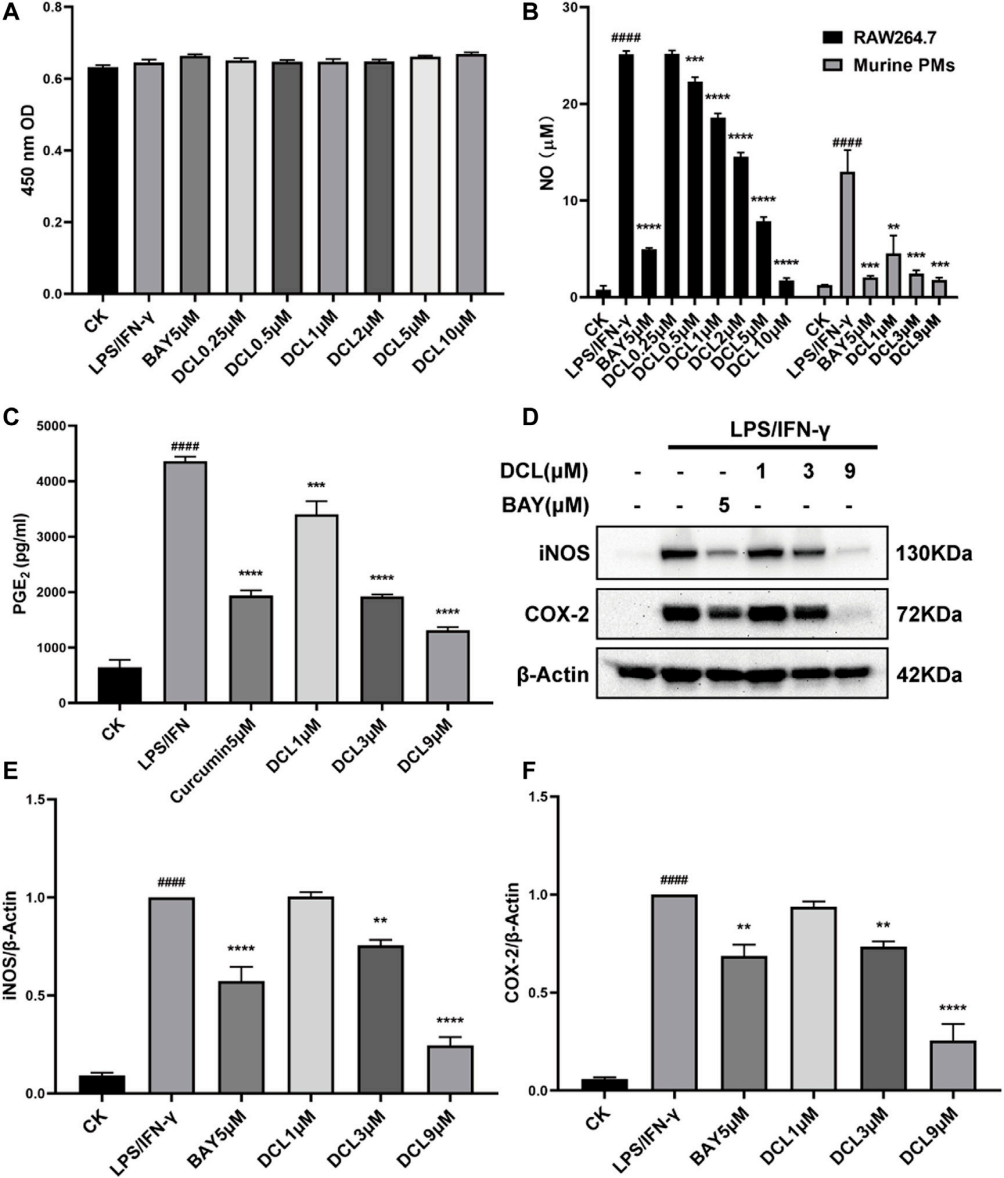
DCL suppresses LPS/IFNγ-induced inflammatory response in murine macrophages. **(A)** Cell viability of LPS/IFNγ-stimulated RAW264.7 cells treated with different concentrations of DCL or BAY was measured by CCK-8 assay. **(B)** The effects of DCL on NO production in LPS (0.5 μg/ml)/IFNγ (10 ng/ml)-stimulated murine RAW264.7 macrophage cell line and murine primary PMs. **(C)** The effect of DCL on LPS/IFNγ-induced PGE_2_ release in RAW264.7 cells. **(D)** Western blot analysis of iNOS and COX-2 from RAW264.7 cells subjected to LPS/IFNγ stimulation and treated with different concentrations of DCL or BAY. The densitometry analysis of iNOS **(E)** and COX-2 **(F)**, normalized against β-Actin. Data are represented as mean ± SEM, *n* = 3. ^#^ *p* < 0.05, ^##^ *p* < 0.01, ^###^ *p* < 0.001, ^####^ *p* < 0.0001 compared to the control check (CK) group. ^*^ *p* < 0.05, ^**^ *p* < 0.01, ^***^ *p* < 0.001, ^****^ *p* < 0.0001 compared to LPS/IFNγ group.

Because macrophages have an irreplaceable role in the pathogenesis of UC, we used LPS/IFNγ-stimulated murine RAW264.7 macrophages to evaluate the anti-inflammatory effect of DCL *in vitro*. DCL treatment inhibited LPS/IFNγ-induced NO production in RAW264.7 cells in a dose-dependent manner without obvious cytotoxicity (Figures 4A,B). The half-inhibitory concentration (IC_50_) of DCL against LPS/IFNγ-induced NO production was 2.283 μM. Furthermore, DCL also reduced NO production in LPS/IFNγ-activated primary mouse peritoneal macrophages in a dose-dependent manner (Figure 4B). In addition, DCL treatment inhibited the release of PGE_2_, another well-known proinflammatory mediator, in LPS/IFNγ-induced RAW264.7 cells (Figure 4C). Because iNOS and COX-2 are responsible for the production of NO and PGE_2,_ respectively, we then detected the protein expression of iNOS and COX-2 in LPS/IFNγ-treated RAW264.7 cells. As expected, the LPS/IFNγ-induced elevated expression of iNOS and COX-2 was significantly blocked upon treatment with different concentrations of DCL or BAY11-7082 (NF-κB inhibitor, 5 μM) (Figures 4D–F). These results suggested that DCL exhibits a potent anti-inflammatory effect in LPS/IFNγ-stimulated RAW264.7 macrophages.

### Dehydrocostus Lactone Blocks the LPS/IFNγ-Induced Activation of NF-κB Signalling in RAW264.7 Macrophages

**FIGURE 5:**
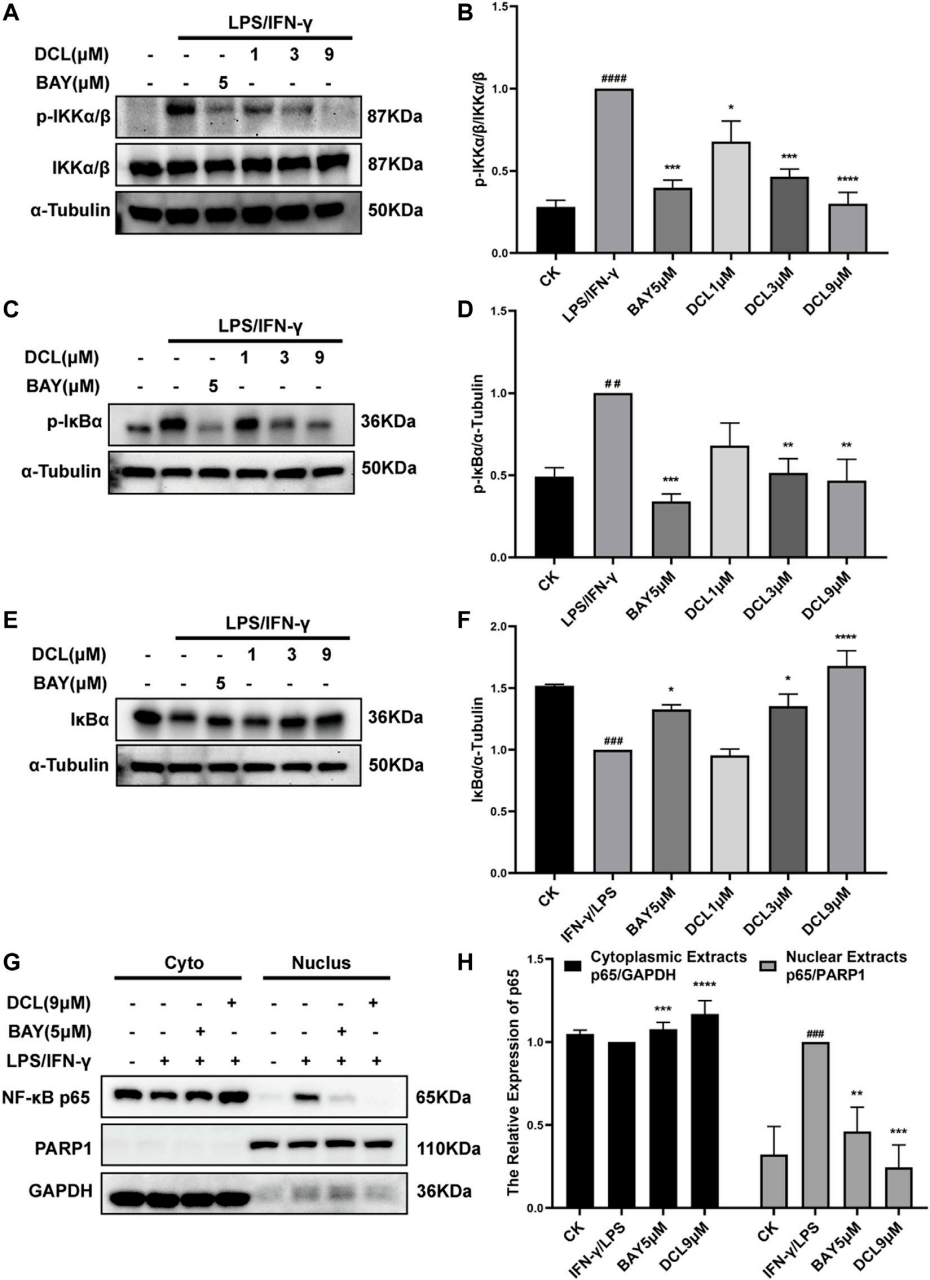
DCL inhibits the activation of NF-κB signalling pathway in the LPS/IFNγ-stimulated macrophages. RAW264.7 cells were treated with DCL (1, 3, and 9 μM) or BAY (5 μM) individually for 2 h, and followed by LPS/IFNγ incubation for 5 min. The protein expression of p-IKKα/β **(A)**, p-IκBα **(C)** and IκBα **(E)** were measured by western blot. The densitometry analysis of p-IKKα/β **(B)**, p-IκBα **(D**) and IκBα **(F)**, normalized against IKKα/β, α-Tubulin respectively. **(G)** The effect of DCL on LPS/IFNγ-induced nuclear translocation of NF-κB p65 in RAW264.7 cells. The densitometry analysis of cytoplasmic and nuclear p65 **(H)**, normalized against GAPDH and PARP1, respectively. Data are represented as mean ± SEM, *n* = 3. ^#^ *p* < 0.05, ^##^ *p* < 0.01, ^###^ *p* < 0.001, ^####^ *p* < 0.0001 compared to CK group. ^*^ *p* < 0.05, ^**^ *p* < 0.01, ^***^ *p* < 0.001, ^****^ *p* < 0.0001 compared to LPS/IFNγ group.

NF-κB is a crucial transcription factor involved in immune and inflammatory processes, and excessive activation of NF-κB plays a critical role at the onset and during the progression of UC (Atreya et al., 2008). Therefore, we investigated whether the anti-inflammatory activity of DCL depends on its regulation of NF-κB signalling. As expected, LPS/IFNγ treatment increased the phosphorylated levels of IKKα/β (Figures 5A,B) and IκBα (Figures 5C,D) but degraded IκBα (Figures 5E,F), whereas DCL or BAY11-7082 (5 μM) treatment blocked the LPS/IFNγ-induced activation of IKKα/β, IKKα/β-mediated phosphorylation and degradation of IκBα in RAW264.7 cells (Figures 5A–F). Notably, DCL at a dose of 3 μM partially blocked LPS/IFNγ-induced IKKα/β phosphorylation (Figures 5A,B). Furthermore, LPS/IFNγ treatment also increased the level of the NF-κB p65 subunit in the nuclear fraction with a corresponding decrease in the cytoplasmic fraction (Figures 5G,H), suggesting activation of NF-κB signalling. DCL or BAY treatment effectively inhibited the LPS/IFNγ-induced nuclear translocation of NF-κB p65 in RAW264.7 cells (Figures 5G,H). Collectively, our results demonstrated that DCL is a potent inhibitor of NF-κB signalling in LPS/IFNγ-stimulated RAW264.7 macrophages.

### Dehydrocostus Lactone Activates Keap1/Nrf2/HO-1 Signalling in LPS/IFNγ-stimulated RAW264.7 Macrophages

**FIGURE 6:**
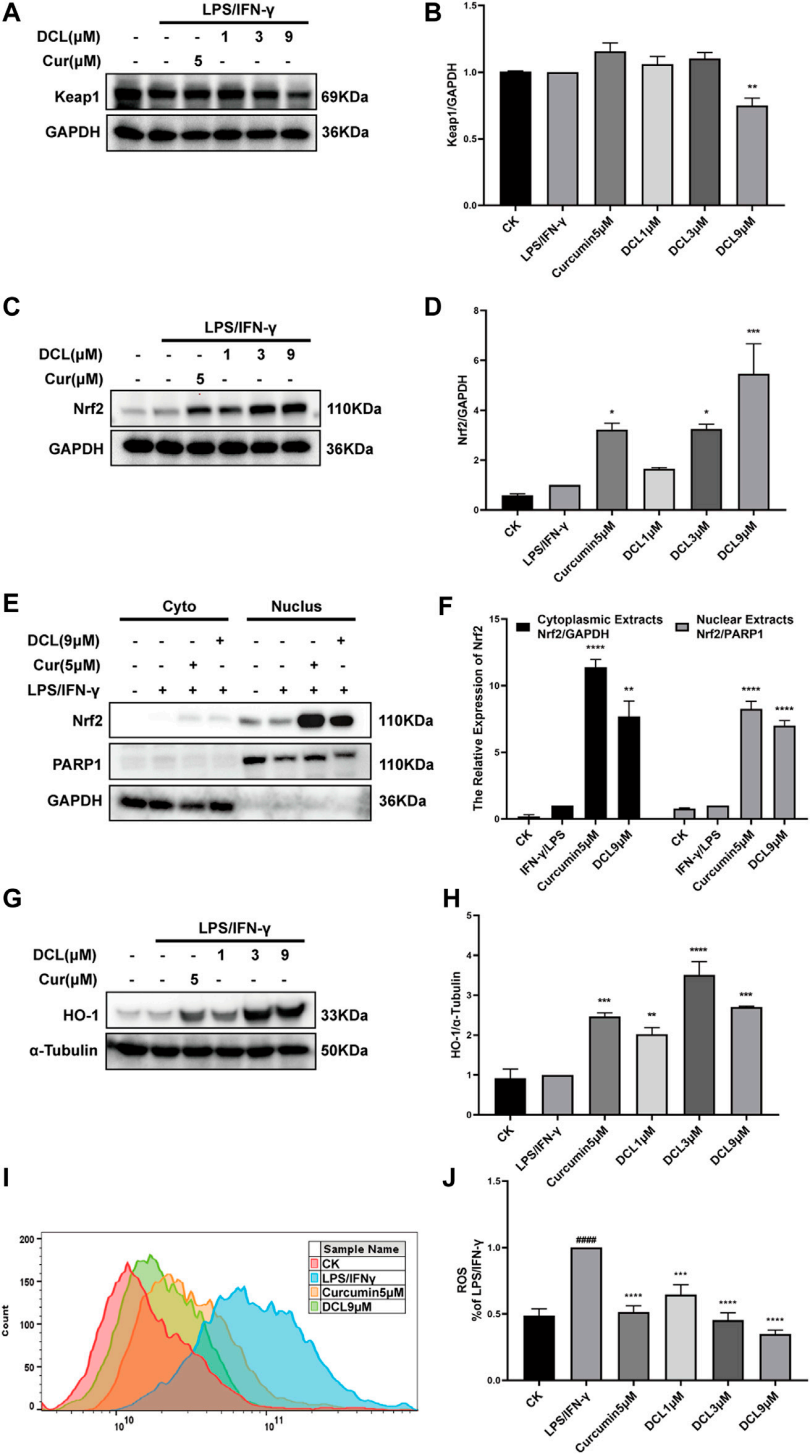
DCL enhances the activation of Keap1/Nrf2/HO-1 in LPS/IFNγ-stimulated RAW264.7 macrophages. RAW264.7 cells were treated with DCL (1, 3, and 9 μM) or curcumin (5 μM) for 1 h, and followed by LPS/IFNγ incubation for additional 8 h. The protein expression of Keap1 **(A)**, Nrf2 **(C)**, and HO-1 **(G)** were measured by western blot. The densitometry analysis of Keap1 **(B)**, Nrf2 **(D)**, and HO-1 **(H)**, normalized against GAPDH or α-Tubulin, respectively. The distribution of Nrf2 in cytoplasmic and nuclear fractions was analyzed using western blotting **(E)**. The densitometry analysis of cytoplasmic and nuclear Nrf2, normalized against GAPDH and PARP1, respectively **(F)**. **(I**,**J)** ROS production was measured using flow cytometry and relative statistical analysis. Data are represented as mean ± SEM, *n* = 3. ^#^ *p* < 0.05, ^##^ *p* < 0.01, ^###^ *p* < 0.001, ^####^ *p* < 0.0001 compared to CK group. ^*^ *p* < 0.05, ^**^ *p* < 0.01, ^***^ *p* < 0.001, ^****^ *p* < 0.0001 compared to LPS/IFNγ group.

Given that the Keap1/Nrf2/HO-1 pathway is known to play a crucial role in the elimination of excess ROS and oxidative stress during inflammatory diseases, such as UC (Wang et al., 2019), we next tested whether DCL inhibits inflammation through the Keap1/Nrf2/HO-1 pathway. As shown in Figures 6A,B, only DCL at a dose of 9 μM slightly reduced the protein expression of Keap1 in LPS/IFNγ-activated RAW264.7 cells. Conversely, DCL treatment increased the protein level of Nrf2 in LPS/IFNγ-stimulated RAW264.7 cells in a dose-dependent manner (Figures 6C,D), and the positive control, curcumin (5 μM), also increased the Nrf2 protein level. The Nrf2 translocation assay showed that DCL or curcumin not only increased the content of Nrf2 protein in the cytoplasmic fractions but also increased the content of Nrf2 protein in the nuclear fractions (Figures 6E,F). Correspondingly, increased expression of HO-1, a Nrf2-dependent antioxidant gene, was observed in DCL- or curcumin-treated RAW264.7 cells (Figures 6G,H). Furthermore, DCL or curcumin treatment markedly inhibited LPS/IFNγ-induced production of ROS in RAW264.7 cells (Figures 6I,J). Taken together, these results showed that DCL effectively activates Keap1/Nrf2/HO-1 antioxidative signalling in LPS/IFNγ-stimulated RAW264.7 cells.

### Dehydrocostus Lactone Moderately Inhibits LPS/IFNγ-Induced Activation of MAPKs in RAW264.7 Macrophages

**FIGURE 7:**
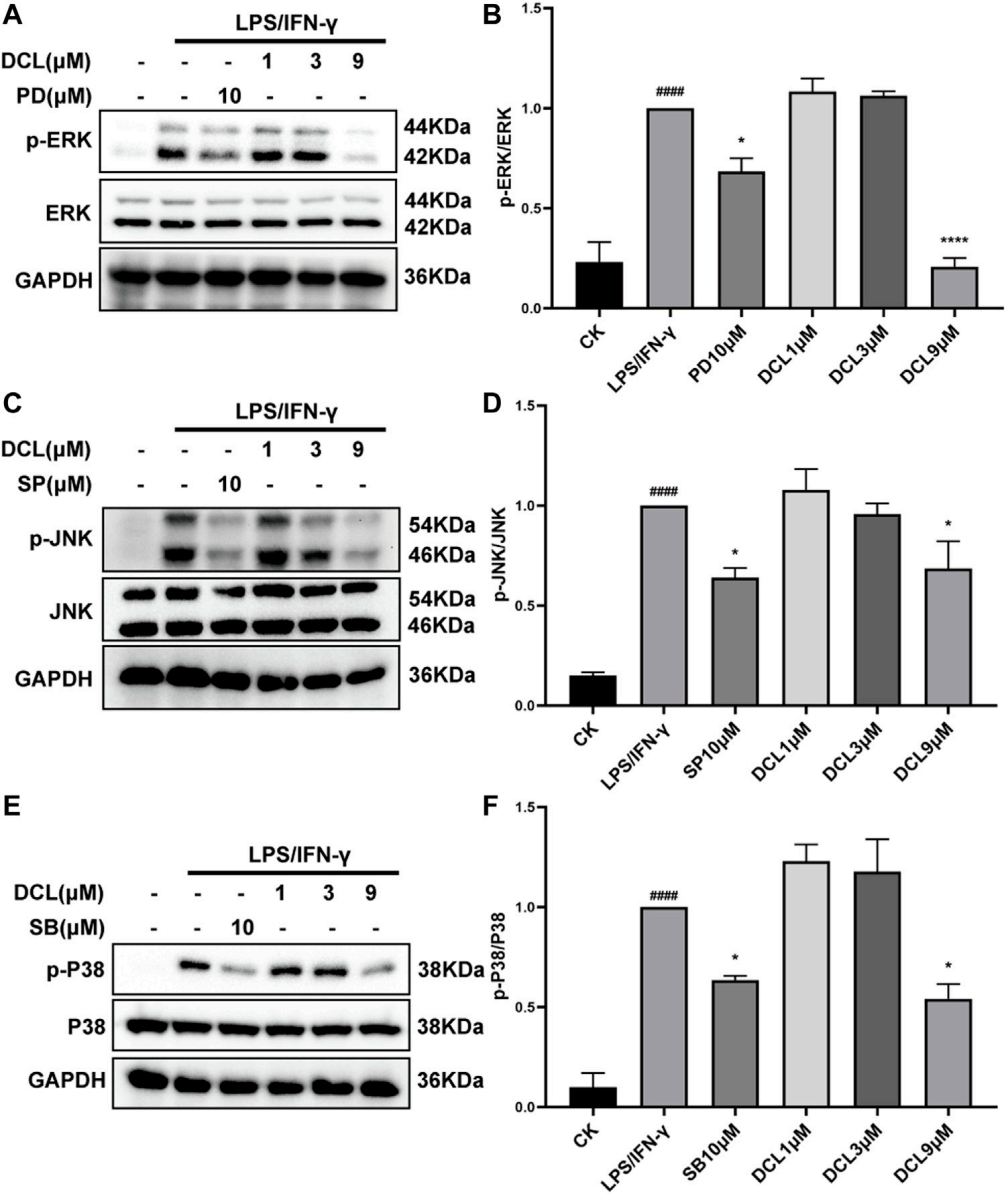
Only 9 μM of DCL decreases the phosphorylation of MAPK in LPS/IFNγ-exposed RAW264.7 macrophages. RAW264.7 cells were treated with DCL (1, 3, and 9 μM), PD (PD98059, 5 μM), SB (SB208530, 5 μM), and SP (SP600125, 5 μM) for 2 h, and subsequently stimulated by LPS/IFNγ for additional 30 min. The phosphorylation of ERK, JNK, and P38 were measured by western blotting. The densitometry analysis of p-ERK **(A)**, p-JNK **(C)**, and p-P38 **(E)**, normalized against total ERK, JNK, and P38 proteins. Data are represented mean ± SEM, *n* = 3. ^#^ *p* < 0.05, ^##^ *p* < 0.01, ^###^ *p* < 0.001, ^####^ *p* < 0.0001 compared to CK group. ^*^ *p* < 0.05, ^**^ *p* < 0.01, ^***^ *p* < 0.001, ^****^ *p* < 0.0001 compared to LPS/IFNγ group.

MAPKs comprise another critical signalling pathway in the regulation of the inflammatory response and have been involved in the pathogenesis of UC (Roy et al., 2008; Zhao et al., 2011). Therefore, we investigated whether DCL affects the activation of the MAPK signalling pathway. Upon LPS/IFNγ stimulation, MAPK pathway proteins, including ERK, JNK, and P38, were significantly phosphorylated in RAW264.7 cells, while PD98059 (ERK inhibitor, 10 μM), SP600125 (JNK inhibitor, 10 μM), and SB203580 (P38 inhibitor, 10 μM) significantly blocked LPS/IFNγ-induced activation of MAPKs (Figures 7A–F). However, DCL at doses of 1 and 3 μM did not inhibit the phosphorylation of MAPKs, whereas 9 μM DCL inhibited the activation of MAPKs in LPS/IFNγ-treated RAW264.7 cells (Figures 7A–F). Taken together, these results revealed that DCL moderately inhibits LPS/IFNγ-induced phosphorylation of MAPKs in RAW264.7 macrophages.

### Dehydrocostus Lactone Interacts With IKKα/β and Keap1

**FIGURE 8:**
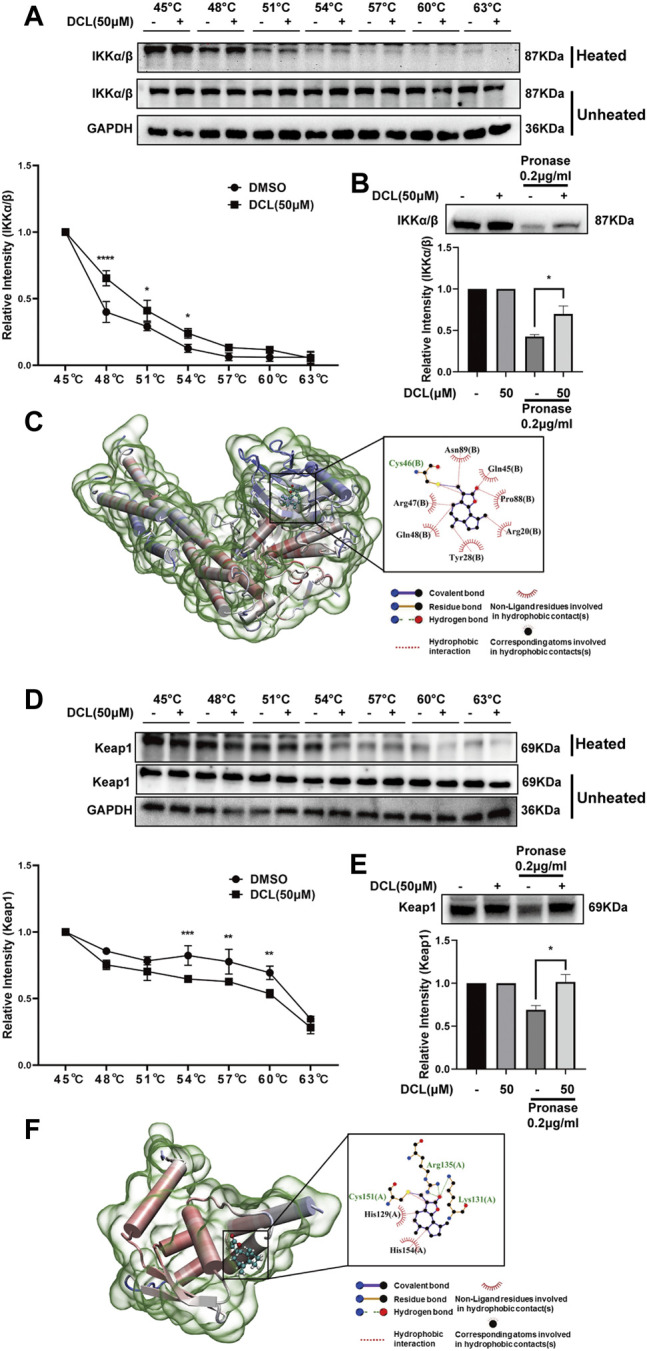
DCL directly interacts with IKKα/β and Keap1 respectively. The effects of DCL on the thermal stability of IKKα/β (**A**, upper panel) and Keap1 (**D**, upper panel) were analyzed by CETSA coupling western blotting assay. The CETSA curves of IKKα/β (**A**, bottom panel) and Keap1 (**D**, bottom panel) were shown. The densitometries of IKKα/β and Keap1 were normalized with respect to those obtained at 45°C. The effects of DCL on the protein’s proteolytic susceptibility of IKKα/β (**B**, upper panel) and Keap1 (**E**, upper panel) were analyzed by DARTS assay. The densitometry analysis of IKKα/β (**B**, bottom panel) and Keap1 (**E**, bottom panel) were shown. Molecular docking of DCL with IKKβ (**C**, PDB: 3QA8) and Keap1 (**F**, PDB: 4CXI). The covalent bonds are shown in purple. Data are represented as mean ± SEM, *n* = 3. ^*^ *p* < 0.05, ^**^ *p* < 0.01, ^***^ *p* < 0.001, ^****^ *p* < 0.0001.

The above results suggested that DCL affects the NF-κB and Keap1/Nrf2 signalling pathways rather than the MAPK pathway. Because IKKα/β are crucial upstream kinases involved in the activation of the NF-κB signalling pathway (Mulero et al., 2019), we explored whether DCL interacts with IKKα/β, resulting in the inhibition of NF-κB signalling. The CETSA results showed that DCL (50 μM) significantly increased the thermal stability of IKKα/β at 48–54°C in RAW264.7 cell lysates (Figure 8A), and the DARTS assay also showed that DCL treatment decreased the protease sensitivity of IKKα/β in RAW264.7 cell lysates (Figure 8B), suggesting a direct interaction of DCL with IKKα/β. Furthermore, DCL possesses an unsaturated lactone moiety, which is a Michael acceptor and may react with the active cysteine of its protein target(s) *via* a covalent bond. Thus, we performed a covalent docking assay to reveal the molecular mechanism of DCL-mediated inactivation of IKKα/β. As shown in Figure 8C, a covalent bond was formed between the C11-C13 unsaturated double bond of DCL and the sulfhydryl of Cys46, an allosteric site of IKKα/β. Furthermore, DCL also formed hydrophobic interactions with several residues, including Tyr28, Gln45, Arg47, Gln48, Pro88, and Asn89.

Because Keap1 is considered a negative regulator of the Nrf2 protein, we investigated whether DCL directly interacts with Keap1, leading to the activation of Nrf2/HO-1 signalling (Piotrowska et al., 2021). The CETSA assay showed that DCL markedly reduced the thermal stability of Keap1 at 54–60°C in RAW264.7 cell lysates (Figure 8D); likewise, the DARTS results showed that proteolysis of Keap1 by the pronase subtilisin was clearly decreased by the presence of DCL (Figure 8E), demonstrating a direct interaction between DCL and Keap1. Furthermore, the docking model of keap1-DCL demonstrated that a covalent bond was formed between the sulfhydryl the Cys151 residue of Keap1 and the C11-C13 unsaturated double bond of DCL (Figure 8F). Two hydrogen bonds were formed between the C12 carbonyl of DCL and two amidogens of Arg135 as well as the amidogen of Lsy131. Moreover, DCL also formed hydrophobic interactions with several residues, including His129, Cys151, and His154 (Figure 8F). Taken together, these results demonstrated that DCL potentially interacts with IKKα/β and Keap1, which inhibits the activation of the NF-κB pathway and activates the Nrf2/HO-1 pathway, ultimately suppressing the inflammatory response.

### DTT Abolishes the Anti-inflammatory Activity of Dehydrocostus Lactone

**FIGURE 9:**
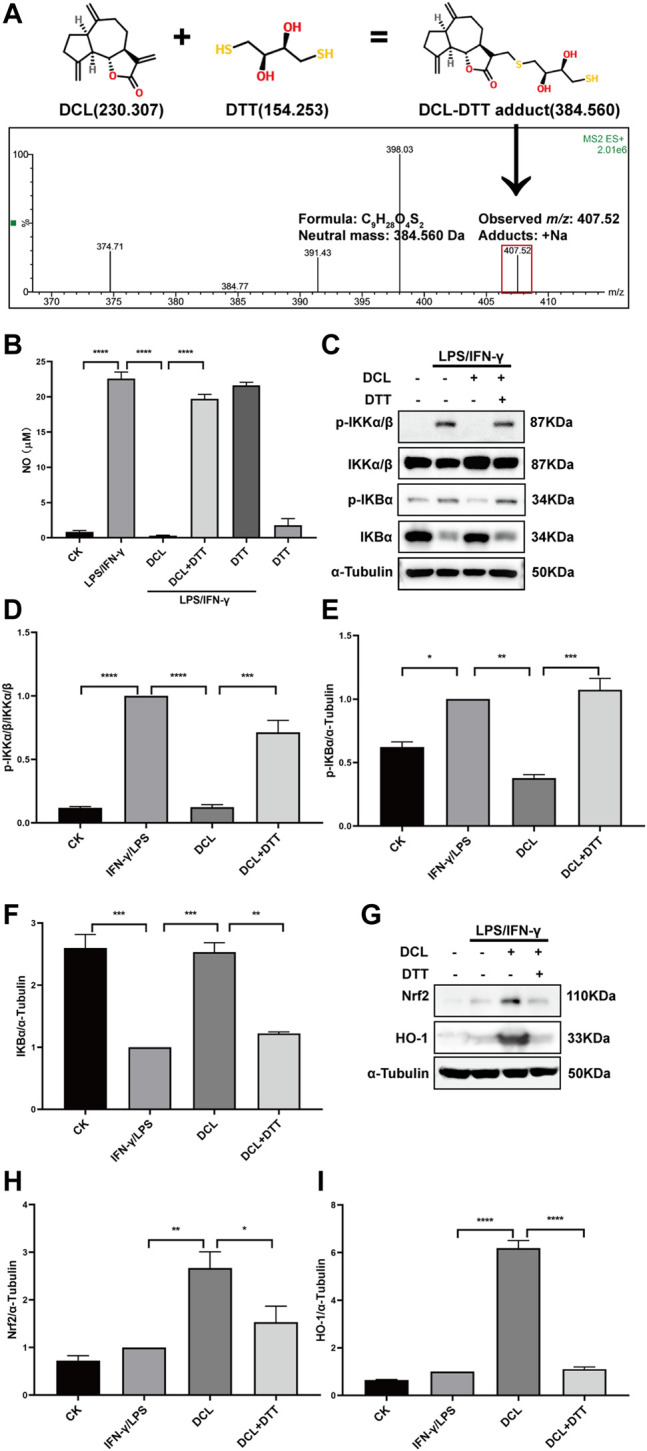
The α-methylene-γ-butyrolactone of DCL is important for its anti-inflammatory effects in LPS/IFNγ-stimulated RAW264.7 cells. **(A)** A possible Michael addition reaction between DCL and DTT is illustrated. The addition product of DCL and DTT was detected by LC-MS assay. **(B)** RAW264.7 cells were stimulated by LPS/IFNγ in the presence of DCL (9 μM) or DCL+DTT (50 μM) for 24 h and then the NO production was analyzed by Griess reagent. RAW264.7 cells were treated with DCL or DCL+DTT for 2 h, and followed by LPS/IFNγ stimulation for 5 min. The expression of p-IKKα/β, p-IκBα, and IκBα were detected by western blotting **(C).** The densitometry analysis of p-IKKα/β **(D)**, p-IκBα **(E)** and IκBα **(F)**, normalized against IKKα/β and α-Tubulin, respectively. RAW264.7 cells were treated with DCL or DCL+DTT for 1 h, and followed by LPS/IFNγ stimulation for 8 h. The expression of Nrf2 and HO-1 were measured by western blotting **(G)**. The densitometry analysis of Nrf2 **(H)** and HO-1 **(I)**, normalized against α-Tubulin. Data are represented as mean ± SEM, *n* = 3. ^*^ *p* < 0.05, ^**^ *p* < 0.01, ^***^ *p* < 0.001, ^****^ *p* < 0.0001.

The unsaturated lactone group of DCL can serve as a Michael acceptor, which covalently binds to the target proteins by reacting with the thiols of proteins. The docking assay also showed that DCL formed covalent bonds with the thiol group of the cysteine in IKKα/β and Keap1. To confirm these results, we performed a LC/MS assay to detect the reaction products of DCL and DTT, an effective thiol ligand donor. The LC/MS results showed that a new addition product at m/z 407.52 [DCL+DTT+Na] was detected (Figure 9A), indicating the covalent interaction between DCL and DTT *in vitro*. Furthermore, we found that DTT-preincubated DCL failed to inhibit LPS/IFNγ-induced NO production in RAW264.7 cells (Figure 9B). Moreover, pretreatment with DTT abolished the inhibitory effect of DCL on the phosphorylation of IKKα/β (Figures 9C,D) and IκBα (Figures 9C,E) as well as the degradation of IκBα (Figures 9C,F) in LPS/IFNγ-stimulated RAW264.7 cells. Similarly, the DCL-induced increased expression of Nrf2 (Figures 9G,H) and HO-1 (Figures 9G,I) was suppressed by DTT pretreatment. Taken together, these results indicated that the α, γ-unsaturated carbonyl group exerts a crucial role in DCL-mediated anti-inflammatory effects by regulating the NF-κB and Keap1/Nrf2/HO-1 signalling pathways.

## Discussion

Given the unsatisfactory effects of traditional therapies for UC, there is an urgent need to identify effective and safe alternative treatments. Accumulating studies have demonstrated that natural products originating from medicinal food homologous plants exhibit beneficial roles in a series of diseases (Hou and Jiang, 2013), including UC (Cai et al., 2019; Han et al., 2021). To evaluate the therapeutic effects of candidates, several chemical-induced colitis models were established. DSS and 2, 4, 6-Trinitrobenzene sulfonic acid (TNBS) are the two most commonly used colitis inducers. Among them, DSS-induced colitis is a classical acute inflammatory model characterized by the dysfunction of the mucosal barrier, while TNBS-induced colitis is a chronic inflammation mediated by T-cell immunity which can maintain a long time (Dong J. et al., 2021; Dong J. Y. et al., 2021). In this study, using DSS-induced colitis model, we found that DCL treatment effectively attenuated DSS-induced colitis, which mainly manifested as weight loss, colon length shortening, inflammatory cell infiltration, and colonic barrier injury. We also demonstrated the key roles of IKKα/β-NF-κB signalling and the Keap1-Nrf2 pathway in DCL-mediated anti-inflammatory action. We also identified the involvement of the MAPK signalling pathway in DCL-induced anti-inflammatory activity.

The major feature of UC is the severe inflammation of the colonic mucosa, which is mainly driven by macrophages. In response to Toll-like receptor (TLR) ligands (such as bacterial LPS) and cytokines (such as IFNγ), intestinal resident macrophages can be activated by a series of intracellular signal transduction pathways (Dorrington and Fraser, 2019). In turn, activated macrophages produce excessive cytokines, chemokines, mediators, and enzymes, which are essential for the triggering, amplification, and maintenance of inflammation, resulting in mucosal damage and barrier dysfunction (Shin and Kim, 2018). In DSS-treated mice, DSS induces mucosal damage and microbial translocation, thereby activating macrophages. As expected, DSS-challenged mice showed significant colon injury and its concomitant immunopathology, including elevated levels of the CD68 macrophage biomarker, elevated levels of proinflammatory cytokines in the lamina propria, an undermined tight junction barrier, and atrophy of the intestinal villi (Lissner et al., 2015). DCL treatment effectively alleviated these abnormal changes, especially the expression of CD68 protein in colon tissues. Therefore, we speculated that the therapeutic effect of DCL on DSS-induced colitis is due, at least in part, to its regulation of the activation and function of macrophages.

Dysregulated production of cytokines and signal transduction by intestinal macrophages have been implicated in the pathogenesis of UC, and NF-κB signalling is one of the major modulators in this complicated condition (Ge et al., 2017). Clinical data have shown elevated expression and activation of NF-κB p65 in the inflamed gut specimens of UC patients, especially in macrophages (Atreya et al., 2008). In UC patients, the increased expression of NF-κB p65 in intestinal mucosal macrophages is accompanied by an elevated capacity of these cells to produce proinflammatory cytokines (Zhou et al., 2018). Furthermore, the degree of activated NF-κB is also positively correlated with the severity of UC (Greten et al., 2004). Currently available anti-UC drugs, such as corticosteroids, 5-ASA, SASP, and methotrexate, exhibit their therapeutic effects at least partly by blocking the NF-κB pathway (Mbodji et al., 2013). Therefore, NF-κB signalling is a promising therapeutic target for the treatment of UC. In the present study, we found that DCL inhibited LPS/IFNγ-induced phosphorylation of IKKα/β and IκBα as well as degradation of IκBα and nuclear translocation of NF-κB p65, suggesting a potent inhibitory role of DCL on NF-κB activation in RAW264.7 macrophages. Although DCL has been shown to inhibit NF-κB signalling in different cell types in diverse cellular contexts (Wang J. et al., 2017), its cellular targets are still unknown. Using the CETSA assay in the present study, we demonstrated that DCL treatment increased the thermal stability of IKKα/β. CETSA is based on the biophysical principle of ligand-induced thermal shift of target proteins, and has been considered a valuable tool for the validation of drug targets (Martinez-Molina et al., 2013; Xiao et al., 2017). Therefore, the addition of DCL increased the thermal stability of IKKα/β suggested a direct interaction between DCL and IKKα/β. Furthermore, we found that DCL significantly inhibited pronase-induced degradation of IKKα/β using DARTS assay. According to the principle of DARTS, ligand binding increases the resistance against proteolysis of the target protein (Lomenick et al., 2011). Thus, these results further support the direct interaction of DCL with IKKα/β. Additionally, molecular docking assays showed that DCL formed a covalent bond with the conserved Cys46 of IKKα/β *via* its C11-C13 unsaturated double bond. A previous study has indicated that Cys46 is an allosteric site of IKKα/β, and covalently modifying Cys46 *via* a small molecule suppresses the activity of IKKα/β, leading to the inhibition of the NF-κB pathway (Dong et al., 2015; Chen et al., 2019). Therefore, we speculated that the covalent binding of DCL to IKKα/β may contribute to the DCL-induced inhibition of the activation of IKKα/β-NF-κB signalling.

In addition to the NF-κB pathway, oxidative stress has been considered to be another early-stage trigger of intestinal inflammation and colon injury (Khor et al., 2006). Nrf2 is a key transcription factor that regulates the expression of a series of antioxidative genes (such as HO-1) in response to tissue oxidative stress (Bellezza et al., 2018). In preclinical studies, activation of Nrf2 signalling has been demonstrated to protect mice against DSS-induced colitis (Rapa et al., 2020; Chen et al., 2021). In the present study, we found that DCL treatment resulted in an accumulation of Nrf2 in the nucleus and induction of HO-1 in LPS/IFNγ-stimulated RAW264.7 cells. Considering the protective effect of Nrf2 signalling in UC therapy, we speculated that the activation of DCL on the Nrf2 pathway may contribute to ameliorating colon injury in DSS-treated mice. Under normal conditions, Keap1 serves as an adaptor for Cullin 3-based E3 ubiquitin ligase and promotes Nrf2 degradation *via* the ubiquitin–proteasome pathway; however, Nrf2 is stabilized when Keap1 is inactivated by oxidative and/or electrophilic stress (Bellezza et al., 2018). Hence, Keap1 is a potential target for Nrf2 activation. In the present study, a high dose of DCL reduced Keap1 protein in LPS/IFNγ-stimulated RAW264.7 cells. Furthermore, the CETSA assay showed that DCL treatment decreased the thermal stability of Keap1; and DARTS assay indicated that DCL treatment reduced protease sensitivity of the Keap1. These results suggested that DCL not only directly interacts with the Keap1 protein but also induces instability and degradation of Keap1 in RAW264.7 macrophages. Moreover, a molecular docking assay showed that the thiol group of the C151 residue of Keap1 was covalently modified by DCL. Keap1 possesses multiple sensor cysteine residues that detect various stress or electrophilic stimuli to cause Nrf2 stabilization. Covalent modification at Cys151 in the BTB domain has been demonstrated to diminish the E3 activity of Cullin 3 by disrupting the interaction of Keap1 and Cul3 (Bellezza et al., 2018; Shin et al., 2020). Therefore, we anticipated that covalent binding to Cys151 by DCL might disturb the interaction between Keap1 and Cul3, thereby resulting in the activation of Nrf2 antioxidative signalling.

Notably, DCL contains an unsaturated lactone moiety, which can serve as a Michael acceptor. Furthermore, the docking assay also showed that DCL formed covalent bonds with the reactive cysteines on IKKα/β and Keap1. Therefore, we hypothesized that DCL possesses the potential to react covalently with the thiol group of cysteine on its target proteins. In the present study, LC–MS analysis detected a new addition product at m/z 407.52 [DCL+DTT+Na], suggesting a possible covalent interaction of DCL with a thiol. Furthermore, several studies have confirmed that the α, γ-unsaturated carbonyl moiety is essential for the anti-inflammatory activity of natural products, such as handelin (Wang L. C et al., 2017) and ainsliadimer A (Dong et al., 2015). The present results showed that the DCL-induced inhibition of LPS/IFNγ-stimulated NO production and NF-κB activation as well as the DCL-induced enhancement of Nrf2 activation were abolished by DTT, which formed a covalent linkage with DCL, possibly through a Michael addition reaction. Therefore, we speculated that the α, γ-unsaturated carbonyl moiety is important for the anti-inflammatory activity of DCL, possibly through covalently binding to the cysteines on IKKα/β and Keap1. Furthermore, compared with the traditional non-covalent agents, covalent agents exhibit several therapeutic advantages, including increased potency and prolonged duration of action (Roskoski, 2021). As a result, in the present, we found that DCL treatment exhibited a better therapeutic effect than SASP, a commercialized non-covalent drug for UC therapy.

The MAPK signalling pathway has been shown to play an important role in the pathogenesis of UC by promoting the release of proinflammatory cytokines and mediators (Vukelić et al., 2020). MAPK inhibitors, especially JNK and P38 inhibitors, have repeatedly demonstrated significant efficacy in experimental colitis models (Hou et al., 2021). Therefore, MAPKs are a potentially relevant target for UC therapy. In the present study, 9 μM DCL inhibited LPS/IFNγ-induced activation of MAPKs in RAW264.7 cells, suggesting that the DCL-induced inhibition of MAPK activation may contribute to its anti-inflammatory and anti-colitis effects. However, lower doses of DCL (1 and 3 μM) exhibited potent effects on the NF-κB and Nrf2 signalling pathways but not the MAPK signalling cascade, suggesting that DCL tends to target the NF-κB and Nrf2 pathways. Furthermore, activation of MAPKs also contributes to the progression of UC *via* the NF-κB-mediated proinflammatory signalling cascade (Roy et al., 2008; Zhao et al., 2011). Thus, the DCL-induced inhibition of MAPKs also contributes to its suppression of NF-κB activation.

## Conclusion

**FIGURE 10:**
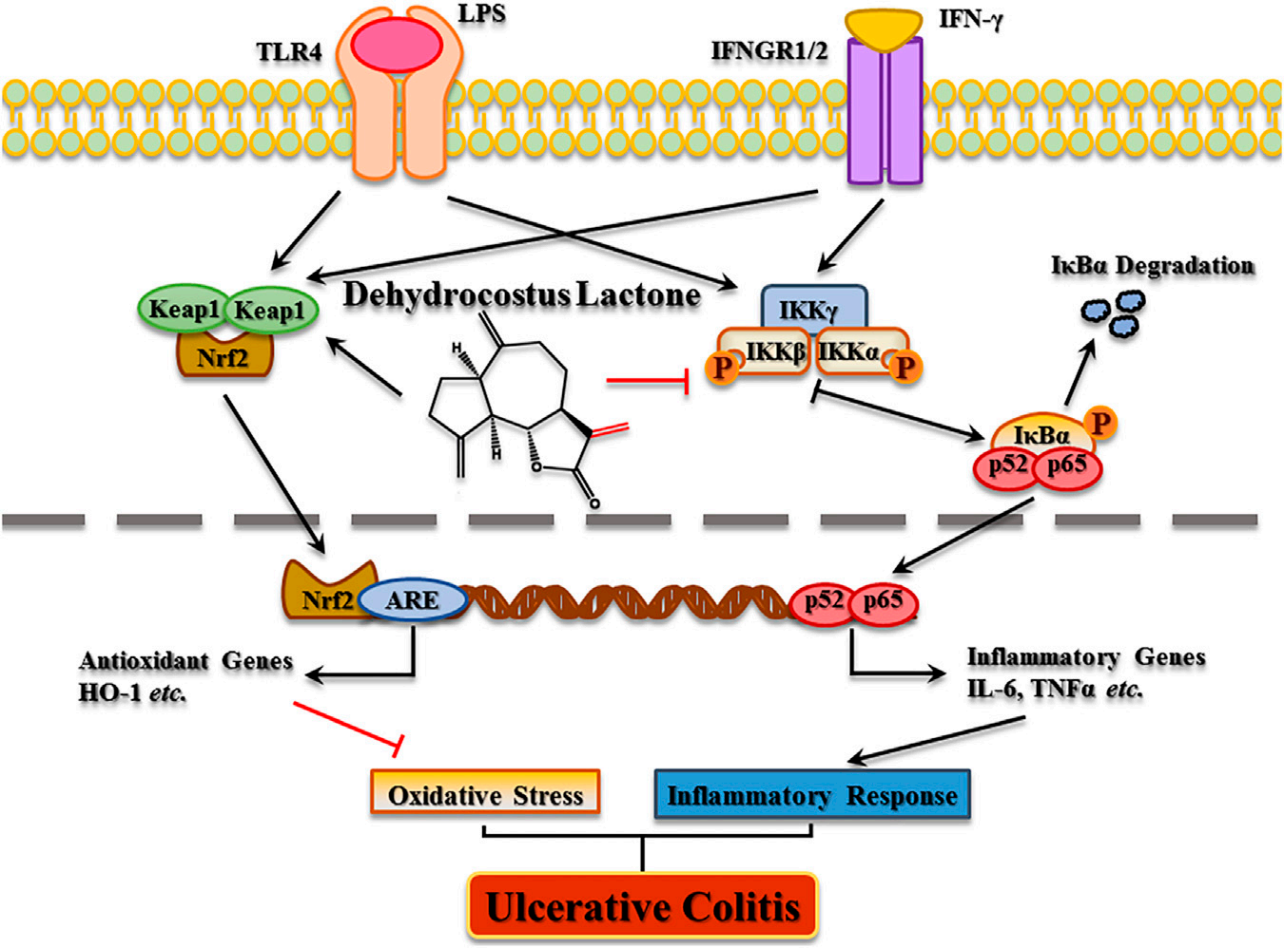
The proposed molecular action model of dehydrocostus lactone (DCL)-mediated anti-inflammatory and anti-oxidative activities in macrophages.

In conclusion, the present study elucidated the molecular mechanism of DCL in experimental colitis. DCL treatment alleviated the progression of DSS-induced colitis through its anti-inflammatory effects by suppressing the NF-κB and MAPK pathways as well as by activating the Nrf2 signalling cascade (Figure 10). Furthermore, IKKα/β and Keap1 were identified as potential targets of DCL-mediated anti-inflammatory action, possibly through the formation of covalent linkages between the cysteines of target proteins and the α, γ-unsaturated carbonyl moiety of DCL. These results suggested that DCL may be an effective therapeutic candidate for UC therapy, but further clinical studies are required.

## Data Availability

The original contributions presented in the study are included in the article, further inquiries can be directed to the corresponding author.

## Glossary

5-ASA,: 5-aminosalicylic acid
BCA,: bicinchoninic acid
CCK-8,: cell Counting Kit-8
CETSA,: Cellular thermal shift assay
DAB,: 3,3′-diaminobenzidine
DARTS,: Drug affinity responsive target stability
DCL,: Dehydrocostus lactone
DSS,: dextran sulfate sodium
ECL,: electrochemiluminescence
FBS,: fetal bovine serum
HE,: hematoxylin and eosin
IBD,: inflammatory bowel diseases
IFNγ,: interferon-γ
Iκ**Bα,**: inhibitor of nuclear factor kappa-B
IKKα/β,: inhibitors of nuclear factor kappa-B kinase α/β
IL-6,: interleukin-6
IOD,: integrated optical density
Keap1,: Kelch-like ECH-associated protein 1
LC-MS,: liquid chromatography-mass spectrometry
LPS,: lipopolysaccharide
MAPK,: mitogen-activated protein kinases
NF-κB,: nuclear factor-κB
NO,: nitric oxide
Nrf2,: nuclear factor erythroid 2-related factor 2
OD,: optical density
PAS,: Periodic acid-schiff
PBS,: phosphate buffer saline
PGE2,: prostaglandin E2
PMs,: peritoneal macrophages
PVDF,: polyvinylidene fluoride
RIPA,: radio immunoprecipitation assay lysis
ROS,: reactive oxygen species
RT,: room temperature
SASP,: sulfasalazine
SDS-PAGE,: sodium dodecyl sulfate-polyacrylamide gel electrophoresis
SEM,: standard error of mean
TLR,: Toll-like receptor
TNF-α,: tumour necrosis factor-α
UC,: ulcerative colitis
